# Management of spontaneous isolated dissection of the superior mesenteric artery: Case report and literature review

**DOI:** 10.1186/1749-7922-6-16

**Published:** 2011-05-08

**Authors:** Morihiro Katsura, Hidemitsu Mototake, Hiroaki Takara, Kazuhide Matsushima

**Affiliations:** 1 Department of General Surgery, Okinawa Prefectural Hokubu Hospital, 2-12-3 Onaka, Nago, Okinawa 905-8512, Japan; 2 Department of Cardiovascular Surgery, Okinawa Prefectural Chubu Hospital, Okinawa, Japan; 3 Department of Radiology, Okinawa Prefectural Chubu Hospital, Okinawa, Japan; 4 Department of Surgery, Penn State Milton S. Hershey Medical Center, PA, USA

## Abstract

**Background and method:**

The aim of this study was to assess retrospectively the clinical presentation, management and outcome of three patients with isolated SMA dissection encountered at Okinawa Prefectural Chubu Hospital, Japan from 2005 to 2006, along with a review of the literature. We follow up the patient's clinical symptoms and the image by using enhanced dynamic CT at 1 week, 1 or 2 months, 6 months, and yearly after onset.

**Case presentation:**

We present three patients with acute abdominal pain due to spontaneous dissection of the superior mesenteric artery (SMA), who were treated by surgical revascularization or conservative management. Two patients underwent surgery because of signs or symptoms of intestinal ischemia and one patient elected conservative management. The SMA was repaired by bypass graft in two cases, and in one of these, the graft was occluded because of prominent native flow from the SMA. All patients were symptom free and there was no evidence of disease recurrence after a median follow-up of 4.3 years.

**Conclusion:**

Although the indications for surgery are still controversial, we should proceed with exploratory laparotomy if the patient has acute symptoms with suspicion of mesenteric ischemia. A non-operative approach for SMA dissection requires close follow-up abdominal CT, with a focus on the clinical signs of mesenteric ischemia and the vascular supply of the SMA, including collateral flow from the celiac artery and inferior mesenteric artery.

## Background

Spontaneous dissection of the superior mesenteric artery (SMA) is not associated with aortic dissection, and is a rare but potentially fatal disease. It is now being reported more often, which is a reflection of the increased use of imaging techniques, such as multidetector row computed tomography (MDCT), multiplanar (MPR) imaging, reconstruction imaging, and CT angiography (CTA) [1-4]. Three different therapeutic approaches are possible: conservative management [5-7], surgical revascularization [8-11], or endovascular therapy [12-18]. However, there is no consensus on the best treatment and its pathogenesis is unclear.

## Case presentation

### Case 1

**Figure 1:**
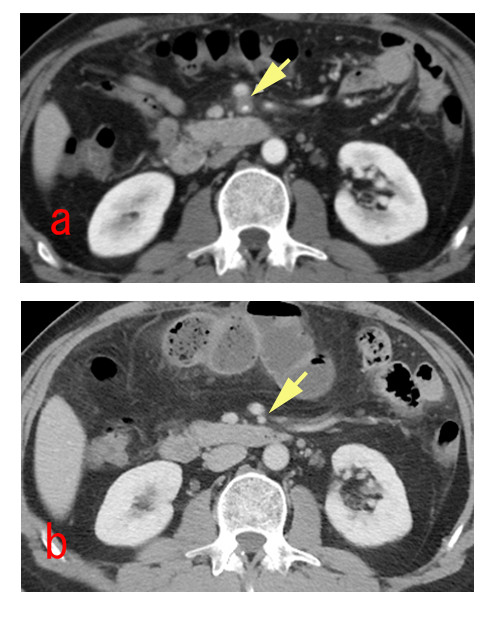
**Sakamoto's type IV dissection of the SMA**. (a) preoperative abdominal enhanced CT scan show isolated dissection of the SMA in which the false lumen was thrombosed without ulcer like projection(ULP). (b) postoperative 1 year abdominal enhanced CT scan show a thrombosed false lumen completely resolved with narrow true lumen.

A 50-year-old man with an 8-day history of epigastric pain of acute onset was admitted. No associated symptoms of fever, nausea, constipation or diarrhea were present. He was previously healthy and had no remarkable medical history and trauma except for hypertension and appendectomy. On physical examination, mild tenderness and rebound tenderness over the epigastrium was observed, and no bruit was audible. Laboratory tests showed slightly elevated serum amylase and bilirubin. Therefore, we initially presumed that the patient had acute pancreatitis, but contrast-enhanced CT revealed isolated dissection of the SMA, in which the false lumen was thrombosed (figure 1a), and the dissecting portion began 6 cm from the origin of the SMA and extended to the distal branch. Bowel ischemia was suspected because of long-term continuous abdominal pain for 8 days and rebound tenderness, even though imaging showed no signs of ischemia. Exploratory laparotomy was performed and revealed a pale and pulseless small bowel without necrosis. We proceeded with a bypass operation between the distal portion of the SMA and the right common iliac artery, using the saphenous vein as a free graft. The postoperative course was uneventful without anticoagulation therapy, and follow-up CT showed good general vascularization of the bowel and full patency of the graft. The patient was discharge on postoperative day 14 and was symptom free 4 years after surgery with no recurrent symptoms or disease progression. One year after surgery, a thrombosed false lumen completely resolved with narrow true lumen on follow up CT(figure 1b).

### Case 2

**Figure 2:**
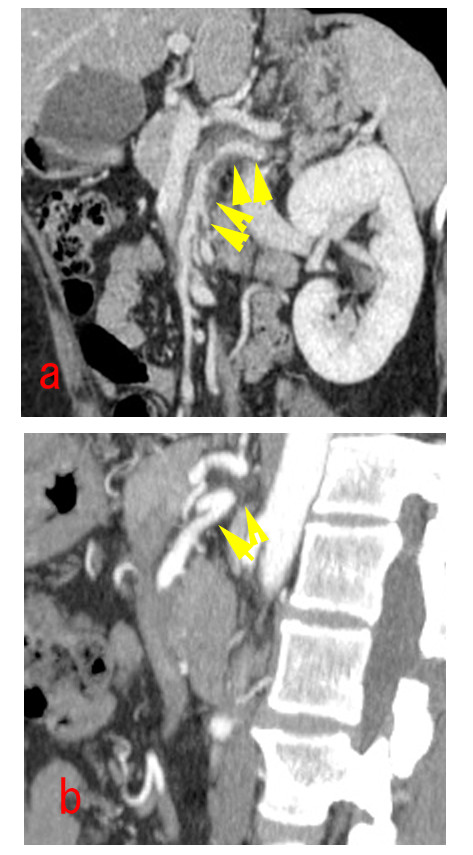
**Sakamoto's type III dissection of the SMA**. (a) preoperative MPR image of an abdominal enhanced CT scan show isolated dissection of the SMA began just after the orifice of the SMA and extended to the distal portion, with ULP and the narrow true lumen being compressed by the thrombosed false lumen. (b) postoperative 3 year abdominal enhanced CT scan show a thrombosed false lumen completely resolved without progressive dilation of ULP.

A 46-year-old woman presented to the emergency department with acute abdominal pain, back pain and vomiting. She had a history of hyperthyroidism but did not have any cardiovascular risk factors or recent trauma. On physical examination, mild periumbilical tenderness without signs of peritonitis was observed. Laboratory tests and abdominal radiography were unremarkable. Contrast-enhanced CT of the SMA showed abnormal wall thickness and irregular diameter, with a double lumen. Isolated dissection of the SMA began from just after the orifice of the SMA and separated the SMA into two distinct lumina for 3 cm from the origin of the artery; the distal portion of the SMA showed signs of thrombosis and stenosis, with the true lumen being compressed by the false lumen (figure 2a). There were no signs of bowel ischemia, such as bowel thickening, abnormal contrast enhancement, or ascites. We proceeded with emergency laparotomy because of continuous severe abdominal pain, but no evidence of ischemia was found throughout the entire bowel with intraoperative duplex scanning. We performed a bypass operation between the distal portion of the SMA and the right common iliac artery, using the saphenous vein as a free graft, to prevent progression of SMA dissection. The postoperative course was uneventful without anticoagulation therapy, but follow-up CT showed thrombotic graft occlusion. We suppose that graft was occluded because of strong native flow from the SMA, that is, flow competition. The patient was discharge on postoperative day 8 and was symptom free 5 years after surgery, with no recurrent symptoms and disease progression. 3 year after surgery, a thrombosed false lumen completely resolved with ulcer like projection (ULP) on follow up CT(figure 2b).

### Case 3

**Figure 3:**
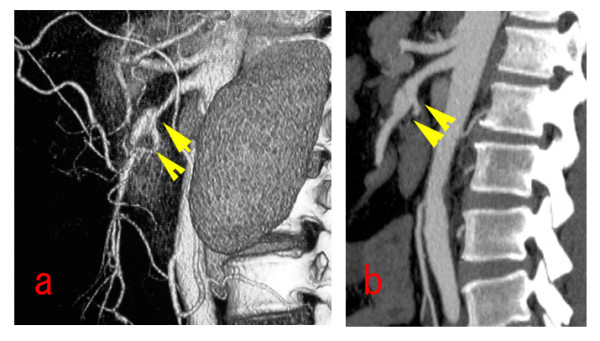
**Sakamoto's type III dissection of the SMA**. (a) preoperative three-dimensionally reconstructed images showing severe stenosis of the SMA with ULP, and the collateral flow from the celiac artery and inferior mesenteric artery. (b) postoperative 1 year abdominal enhanced CT scan show a thrombosed false lumen completely resolved without progressive dilation of ULP.

A 47-year-old man with a 5-day history of acute epigastric pain with radiation to the back was admitted. No associated symptoms of fever, nausea, constipation or diarrhea were present. He was previously healthy and had no cardiovascular risk factors and recent trauma. On physical examination, mild tenderness over the epigastrium without signs of peritonitis sign was observed, and no bruit was audible. Laboratory tests and abdominal radiography were unremarkable. Contrast-enhanced CT revealed a thin flap of the SMA, which began from just after the orifice of the SMA and separated the SMA into two distinct lumina; the resulting false lumen was thrombosed in the mid to distal portion of the SMA. Three-dimensionally reconstructed images demonstrated severe stenosis of the SMA, but no sign of bowel ischemia caused by prominent collateral flow from the celiac artery and inferior mesenteric artery (figure 3a). We chose conservative treatment without anticoagulation therapy. The abdominal pain completely disappeared on day 2 and he was discharged on day 4. The patient was symptom free 4 years after discharge with no recurrent symptoms and disease progression. One year after surgery, a thrombosed false lumen completely resolved with ULP on follow up CT (figure 3b).

## Discussion and review of the literature

Spontaneous dissection of the SMA is a rare condition and is not associated with aortic dissection. It was first described by Bauerfield in 1947 [19]. In previously reported cases before 1972, the prognosis was very poor [19,20]. However, the prognosis has improved significantly since 1975 as a result of advancements in surgical techniques and imaging modalities [1-4].

The etiology of the disease has not yet been established, but atherosclerosis, cystic medial necrosis, and fibromuscular dysplasia have been implicated, often associated with untreated hypertension [3]. Solis et al. [21] have hypothesized that dissection usually begins 1.5-3 cm from the orifice of the SMA, thus sparing the origin of the artery. This segment of the SMA corresponds with the exit of the artery from the pancreas and is exposed to shearing force because this area forms the border zone between the fixed retropancreatic portion and the more distal mobile mesenteric portion. In two of the three present cases, dissection began from just after the orifice of the SMA, and in the other, dissection began 6 cm from the orifice. Our findings were not consistent with the hypothesis of Solis et al., but we suppose that the dissection plane can extend not only distally but also proximally.

The natural history of the disease is also unclear and depends on each case. Most patients present with acute epigastric pain, which is considered to be caused by the dissection itself or intestinal ischemia. Other common symptoms are nausea, vomiting, melena, and abdominal distention. These patients present acutely with symptom duration of <4 weeks [22]. Laboratory tests and abdominal radiography are usually unremarkable. Therefore, we often initially presume that the patient has enterocolitis and gastritis. Sometimes, laboratory tests show slightly elevated serum amylase, such as in our case 1, which might be caused by occlusion of the duodeno-pancreatic arcade [10].

Diagnosis in the acute stage has become possible as a result of advances and increased use of imaging techniques such as MDCT, leading to MPR and reconstruction imaging, and CTA [1-4]. Dynamic enhanced CT shows that the separated true lumen and false lumen can be identified by the presence of an intimal flap. Plain CT shows areas of high intensity if there is an acute clot in the false lumen. Sakamoto et al. [23] have categorized SMA dissection into four types based on contrast-enhanced CT scanning. Recently, Yun et al. [24] have added total thrombotic occlusion of the SMA trunk to Sakamoto's classification, and have devised a new classification of three types based on angiographic findings: type I: patent true and false lumina that show entry and re-entry sites; type II: patent true lumen but no re-entry flow from the false lumen; type IIa: visible false lumen but no visible re-entry site (blind pouch of false lumen); type IIb: no visible false luminal flow (thrombosed false lumen), which usually causes true luminal narrowing; and type III: SMA dissection with occlusion of SMA.

However, neither Sakamoto et al. nor Yun et al. have found a clear relationship between radiological appearance and clinical course. Abdominal color Doppler echo is also effective for following hemodynamic changes within the SMA, bowel movement, and signs of bowel ischemia, such as wall thickening and intestinal dilatation.

Some treatment algorithms for management of spontaneous SMA dissection have been reported [22,25,26]. At present, however, there is no established opinion on the indications for surgical revascularization, conservative medical management, or endovascular therapy. Some cases have been successfully treated by conservative therapy, such as anticoagulation [5,6]. Karacagi et al have reported that immediate anticoagulation therapy achieved prevention of clot formation in the true lumen in patients with spontaneous dissection of the carotid artery[27]. Nagai et al insisted that the disease pattern of SMA dissection seems similar with internal carotid artery and emphasized anticoagulation therapy is necessary for SMA dissection[5]. On the other hand, Sparks et al. [28] have reported a case in which the patient developed recurrent symptoms and disease progression 1 year later, which was a failure of the non-operative approach. This case indicates that a non-operative approach with anticoagulation of the isolated SMA dissection requires close follow-up, but it does not prevent disease progression. At that time, there is no consensus on the best drugs to be administered and administration period, so we didn't give anticoagulant for our case No.3. But we now suppose that anticoagulation therapy is valid for this disease when we chose conservative treatment.

Sparks et al. have suggested that indications for surgery are increasing size of the aneurysmal dilatation of the SMA, luminal thrombosis, or persistent symptoms despite anticoagulation. Various procedures for surgical intervention have been reported [8-11], including aortomesenteric or iliomesenteric bypass, thrombectomy, intimectomy with or without patch angioplasty, ligation, and resection. These surgical procedures have been performed with good short-term results.

Recent minimally invasive techniques, such as percutaneous endovascular stent placement and intralesional thrombolytic therapy, could be useful in certain cases, especially in patients at high risk for surgery [12-18]. However, it is usually difficult to find the site at which tearing of the artery wall started during dissection of the SMA, and the dissection often extends to the distal portion of the SMA, as in our present cases. There are still many problems with stent placement itself, such as risk of re-occlusion of a stented SMA and possible obstruction of side branches of the stented segment. Although we think that endovascular stent placement is feasible in patients without peritonitis or mesenteric ischemia, the long-term results should continue to be evaluated. Intralesional thrombolytic therapy with urokinase have also been reported, but some cases later underwent stenting [13] and laparotomy [29,30] because of clinical deterioration.

Table 1 summarizes the clinical characteristics of our three cases. In the patient whose small intestine we revascularized using an iliac-mesenteric bypass, because of bowel ischemia, postoperative follow-up CT showed good general vascularization of the bowel and full graft patency. On the other hand, in the patient whose small intestine we revascularized to prevent disease progression, although there was no sign of bowel ischemia, postoperative follow-up CT showed thrombotic graft occlusion. We suppose that graft was occluded because of prominent native flow of the SMA, that is, flow competition. Our colleague Matsushima also has reported a case of SMA dissection [31]. In that case, emergency laparotomy was undertaken because the patient had signs that were suspicious of mesenteric ischemia. However, at the time of surgery, no evidence of ischemia was found throughout the entire bowel using intraoperative duplex scanning, which detected adequate blood flow to the peripheral branches, therefore, vascular reconstruction was not performed. Postoperatively, anticoagulants were administered and the patient was free of abdominal symptoms a few days later. We now suppose that it is not necessary to perform vascular reconstruction to prevent disease progression. Conservative management should have been indicated for our case No.2. If a initial CT demonstrated ULP, which was seen in the case like Sakamoto's classification type long term follow up are necessary for recognition of progressive dilation of ULP and aneurismal formation.

**Table 1:** Clinical characteristics of patients with SMA dissection

Case	Age/Sex	Dissection portion	Sakamoto's	Treatment	intestinal ischemia	Follow up CT
**No**.			classification		on surgery	
1	50/M	6 cm from the orifice	type IV	Surgery	Yes	Graft patent
		of the SMA				ULP (-)
2	46/F	just after the orifice	type III	Surgery	None	Graft occlusion
		of the SMA				ULP (+)
3	47/M	just after the orifice	type III	Conservative	-	resolved false lumen
		of the SMA				ULP (+)

ULP: ulcer like projection

## Conclusions

There is no consensus on the best treatment of spontaneous isolated dissection of the SMA. Although the indications for surgery are still controversial, we should proceed with exploratory laparotomy if the patient has acute symptoms with suspicion of mesenteric ischemia. A non-operative approach for SMA dissection requires close follow-up abdominal CT, with a focus on the clinical signs of mesenteric ischemia and the vascular supply of the SMA, including collateral flow from the celiac artery and inferior mesenteric artery.

## Competing interests

The authors declare that they have no competing interests.

## Authors' contributions

All authors except HT were involved in the preoperative and postoperative care of the patient. MK is the primary author and reviewed the case and the literature. HM and KM participated in the surgeries and provided editorial commentary. HT performed the angiography treatment. All authors conceived of the study, and participated in its design and coordination and helped to draft the manuscript. All authors have read and approved the final manuscript.

## References

[B1] Suzuki S Furui S Kohtake H Sakamoto T Yamasaki M Furukawa A Murata K Takei R Isolated dissection of the superior mesenteric artery: CT findings in six cases Abdom Imaging 2004 29 153 157 10.1007/s00261-003-0110-2 15290937

[B2] Hyodoh H Hyodoh K Takahashi K Yamagata M Kanazawa K Three-dimensional CT imaging of an isolated dissecting aneurysm of the superior mesenteric artery Abdom Imaging 1996 21 515 516 10.1007/s002619900116 8875874

[B3] Sheldon PJ Esther JB Sheldon EL Sparks SR Brophy DP Oglevie SB Spontaneous dissection of the superior mesenteric artery Cardiovasc Intervent Radiol 2001 24 329 331 10.1007/s00270-001-2565-0 11815839

[B4] Furukawa H Moriyama N Spontaneous dissection of the superior mesenteric artery diagnosed on multidetector helical CT J Comput Tomogr 2002 26 143 144 10.1097/00004728-200201000-00025 11801921

[B5] Nagai T Torishima R Uchida A Nakashima H Takahashi K Okawara H Oga M Suzuki K Miyamoto S Sato R Murakami K Fujioka T Spontaneous dissection of the superior mesenteric artery in four cases treated with anticoagulation therapy Intern Med 2004 43 473 478 10.2169/internalmedicine.43.473 15283182

[B6] Takayama H Takeda S Saitoh SK Hayashi H Takano T Tanaka K Spontaneous isolated dissection of the superior mesenteric artery Intern Med 2002 41 713 716 10.2169/internalmedicine.41.713 12322798

[B7] Cho YP Ko GY Kim HK Moon KM Kwon TW Conservative management of symptomatic spontaneous isolated dissection of the superior mesenteric artery Br J Surg 2009 96 720 723 10.1002/bjs.6631 19526615

[B8] Kochi K Orihashi K Murakami Y Sueda T Revascularization using arterial conduits for abdominal angina due to isolated and spontaneous dissection of the superior mesenteric artery Ann Vasc Surg 2005 19 418 420 10.1007/s10016-005-0018-0 15834681

[B9] Tsuji Y Hino Y Sugimoto K Matsuda H Okita Y Surgical intervention for isolated dissecting aneurysm of the superior mesenteric artery: A case report Vasc Endovasc Surg 2004 38 469 472 10.1177/153857440403800513 15490047

[B10] Picquet J Abilez O Pénard J Jousset Y Rousselet MC Enon B Superficial femoral artery transposition repaire for isolated superior mesenteric artery dissection J Vasc Surg 2005 42 788 791 10.1016/j.jvs.2005.05.048 16242570

[B11] Cormier F Ferry J Artru B Wechsler B Cormier JM Dissecting aneurysms of the main trunk of the superior mesenteric artery J Vasc Surg 1992 15 424 30 1735904

[B12] Leung DA Schneiber E Kubik-Huch R Marineck B Pfammatter T Acute mesenteric ischemia caused by spontaneous isolated dissection of the superior mesenteric artery: treatment by percutaneous stent placement Eur Radiol 2000 10 1916 1919 10.1007/s003300000520 11305570

[B13] Yoon YW Choi D Cho SY Lee DY Successful treatment of isolated spontaneous superior mesenteric artery dissection with stent placement Cardiovasc Intervent Radiol 2003 26 475 478 10.1007/s00270-003-0002-4 14753308

[B14] Froment P Alerci M Vandoni RE Bogen M Gertsch P Galeazzi G Stenting of a spontaneous dissection of the superior mesenteric artery:a new therapeutic approach? Cardiovasc Intervent Radiol 2004 27 529 532 10.1007/s00270-003-0158-y 15461979

[B15] Kim JH Roh BS Lee YH Choi SS So BJ Isolated spontaneous dissection of the superior mesenteric artery: percutaneous stent placement in two patients Korean J Radiol 2004 5 134 138 10.3348/kjr.2004.5.2.134 PMC2698142 15235239

[B16] Miyamoto N Sakurai Y Hirokami M Takahashi K Nishimori H Tsuji K Kang JH Maguchi H Endovascular stent placement for isolated spontaneous dissection of the superior mesenteric artery: Report of a case Radiat Med 2005 23 520 524 16485545

[B17] Casella IB Bosch MA Sousa WO Jr Isolated spontaneous dissection of the superior mesenteric artery treated by percutaneous stent placement: case report J Vasc Surg 2008 47 197 200 10.1016/j.jvs.2007.07.051 18178474

[B18] Gobble RM Brill ER Rockman CB Hecht EM Lamparello PJ Jacobowitz GR Maldonado TS Endovascular treatment of spontaneous dissections of the superior mesenteric artery J Vasc Surg 2009 50 1326 1332 10.1016/j.jvs.2009.07.019 19782510

[B19] Bauerfield SR Dissecting aneurysm of the aorta:a presentation of fifteen cases and a review of the recent literature Ann Intern Med 1947 26 873 889 10.7326/0003-4819-26-6-873 20242656

[B20] Hirai S Hamanaka Y Mitsui N Isaka M Kobayashi T Spontaneous dissection of the main trunk of the superior mesenteric artery Ann Thorac Cardiovasc Surg 2002 8 236 240 12472390

[B21] Solis MM Ranval TJ McFarland DR Eidt JF Surgical Treatment of superior mesenteric artery dissection aneurysm and simultaneous celiac artery compression Ann Vasc Surg 1993 7 457 462 10.1007/BF02002130 8268091

[B22] Subhas G Gupta A Nawalany M Oppat WF Spontaneous isolated superior mesenteric artery dissection: a case report and literature review with management algorithm Ann Vasc Surg 2009 23 788 798 10.1016/j.avsg.2008.12.006 19467833

[B23] Sakamoto I Ogawa Y Sueyoshi E Fukui K Murakami T Uetani M Imaging appearances and management of isolated spontaneous dissection of the superior mesenteric artery Eur J Radiol 2007 64 103 110 10.1016/j.ejrad.2007.05.027 17628380

[B24] Yun WS Kim YW Park KB Cho SK Do YS Lee KB Kim DI Kim DK Clinical and angiographic follow-up of spontaneous isolated superior mesenteric artery dissection Eur J Vasc Endovasc Surg 2009 37 572 577 10.1016/j.ejvs.2008.12.010 19208448

[B25] Morris JT Guerriero J Sage JG Mansour MA Three isolated superior mesenteric artery dissections: update of previous case reports, diagnostics, and treatment options J Vasc Surg 2008 47 649 653 10.1016/j.jvs.2007.08.052 18295120

[B26] Zerbib P Perot C Lambert M Seblini M Pruvot FR Chambon JP Management of isolated spontaneous dissection of superior mesenteric artery Langenbecks Arch Surg 2010 395 437 443 10.1007/s00423-009-0537-1 19588161

[B27] Karacagil S Hardemark HG Bergqvist D Spontaneous internal carotid artery dissection Int Angiol 1996 15 291 294 9127767

[B28] Sparks SR Vasquez JC Bergan JJ Owens EL Failure of nonoperative management of isolated superior mesenteric artery dissection Ann Vasc Surg 2000 14 105 109 10.1007/s100169910019 10742422

[B29] Javerliat I Becquemin JP d'Audiffret A Spontaneous isolated dissection of the superior mesenteric artery Eur J Vasc Endovasc Surg 2003 25 180 184 10.1053/ejvs.2002.1785 12552483

[B30] Hwang CK Wang JY Chaikof EL Spontaneous dissection of the superior mesenteric artery Ann Vasc Surg 2010 24 254.e1 5 10.1016/j.avsg.2009.09.013 20142003

[B31] Matsushima K Spontaneous isolated dissection of the superior mesenteric artery Am Coll Surg 2006 203 970 971 10.1016/j.jamcollsurg.2006.03.018 17116566

